# Effect of Medicaid Coverage of Tobacco-Dependence Treatments on Smoking Cessation

**DOI:** 10.3390/ijerph6123143

**Published:** 2009-12-09

**Authors:** Feng Liu

**Affiliations:** School of Economics, Shanghai University of Finance and Economics, 777 Guoding Road, Shanghai 200433, China; E-Mail: liu.feng@mail.shufe.edu.cn; Tel.: +86-21-6590-2331; Fax: +86-21-6590-4198

**Keywords:** Medicaid, smoking cessation, health insurance, tobacco dependence treatment

## Abstract

Smoking cessation aids (nicotine replacement products and anti-depressant medication) have been proven to double quitting rates compared to placebo in several randomized controlled trials. But the high initial cost of cessation aids might create a financial barrier to cessation for low-income smokers. In the U.S., Medicaid provides health insurance coverage to low-income people, and in some states covers smoking cessation products. This paper uses nationally representative data of the U.S. to examine how the Medicaid coverage of cessation aids affect smoking behavior. The results indicate the Medicaid coverage of cessation products is positively associated with successful quitting among women aged 18–44.

## 1. Introduction

According to the Current Population Survey (CPS)—Tobacco Usage Supplement (TUS) 1992–2003 in the U.S. 26% of Medicaid recipients (age 15 to 90) are everyday smokers, compared to 16% for the general population. The probability of smoking among Medicaid recipients is approximately 50% higher than among the non-Medicaid population.

Most smokers want to quit. CPS-TUS data reveal that 73% current smokers have ever tried to quit for at least one day. In a typical year, 45% of smokers try to quit smoking. However, fewer than 5% succeed in quitting each year [1]. Smokers can take Tobacco Dependence Treatments (TDT) to increase quitting success rates. There are two basic forms of TDT: pharmacotherapy treatments (nicotine replacement products and anti-depressant medication) and counseling service. A meta-analysis study [2] suggests nicotine replacement therapy (NRT) doubles successful quitting rates. Other studies [3,4] have shown evidence that nicotine replacement therapy, in combination with counseling services is more successful than drug therapy alone. Counseling services may include stress management, weight control, tips for preventing relapse, and other behavioral or psychological support.

But TDT costs money. Among smokers who have never used any NRT products, cost is the most frequently cited reason [5]. For example, a 30 day supply of skin patches will cost about $112. Since Medicaid recipients have low incomes, the high initial cost of treatments might create a barrier to using TDT for cessation [6]. Providing coverage of TDT would overcome the financial barrier for smokers interested in quitting and possibly help to increase successful smoking cessation rate.

This study applies a nationally representative large dataset to examine in detail how Medicaid coverage of TDT affects smoking cessation. First, a descriptive analysis examines whether usage of TDT among Medicaid recipients differs in the states with and without TDT coverage. Second, the sample is divided into four groups by age and gender, and separate regression is run for each group to investigate differences in smokers’ responsiveness to TDT coverage. Finally, state fixed effects models are estimated to control for unobserved differences across states. The study also adopts an alternative approach by using state anti-smoking sentiment index explicitly to solve potential multicollinearity problem in state fixed effects models.

## 2. Medicaid Background

Medicaid was established in 1965 to provide health care service to low-income American families. There are three kinds of Medicaid recipients: low-income children and women; the disabled; and the low-income elderly. Each state administers its own programs under broad federal guidelines. Medicaid is jointly financed by the federal and state governments. The federal matching rate depends on state's average per capita income level. Federal government reimburses states at a lower rate if the state has higher per capita income. By law, federal matching rate is between 50% and 83%. In 2002, the average matching rate of federal funds is 57% overall [7]. Smoking-caused Medicaid costs have increased from $12.9 billion in 1993 to $27.2 billion in 2001 [8,9]. According to National Commission on Prevention Priorities, smoking is one of the three preventive services that are cost saving [10].

By federal rules, states may choose whether or not to cover the costs of TDT. Each state might have different measures of cost and benefit before deciding whether offering coverage of TDT. The costs include pretreatment screening to identify smokers, advising or motivating smokers to quit, actual cost of NRT products, costs of physicians, nurses and counseling providers. Most benefits from smoking cessation occur over a long period of time, like reducing the risks of developing lung cancer, heart attack and stroke. In the short-run, benefits include decrease of coughing and shortness of breath [11]. For pregnant women, smoking cessation leads to fewer low birth weight infants. Consequently, health care expenditures will be reduced.

Lawmakers from tobacco-growing states (Kentucky, North Carolina, Tennessee, and Virginia) are less likely to vote in favor of tobacco control legislation because doing so might hurt the economy of these states or just because of political reasons—they do not want to alienate important political constituencies—tobacco farmers and others employed in the tobacco industry. Flynn *et al*. [12] find that legislators in Vermont were almost 21 times more likely to intend to vote for cigarette tax increases than lawmakers in North Carolina. Similarly, legislators in Kentucky did not consider covering the smoking cessation aids by the state Medicaid plan until 2006 [13].

## 3. Literature Review

Several studies have examined the effect of insurance coverage of TDT on smoking cessation. Curry *et al*. [14] compared the cessation behavior of smokers who enrolled in four different insurance plans in Washington State. The four plans comprised of different combinations of co-insurance: (1) 50% coverage of both behavioral programs and nicotine-replacement therapy (NRT); (2) 50% coverage of behavioral programs and full coverage of NRT; (3) full coverage of behavioral programs and 50% coverage of NRT; 4) full coverage of both behavioral programs and NRT. They find out, on average, 2.8% smokers under full coverage quit smoking per year, compared to 1.9% smokers quitting under the cost-sharing plans.

Boyle *et al*. [15] studied the effect of a new health plan in Minnesota covering NRT (gum and patch) and Zyban^®^ (an anti-depressant medication) on the use of these products and quitting rate. They find that smokers in plans that covered the cost of smoking-cessation pharmacy products were no more likely to quit than those in plans without the benefit. One possible reason is the knowledge gap as the authors find only 30% of the smokers whose plan included the benefit reported knowing it. Similarly, a report by the Centers for Disease Control and Prevention [16] finds that only 28% of states that offer TDT coverage to Medicaid recipients inform their beneficiaries of these benefits.

Schauffer *et al*. [17] conducted a randomized experiment among smokers enrolled in HMOs in California. The nicotine patch, gum, and group counseling are covered in the treatment group. They find the quitting rate among smokers in treatment group was 18% over 12 months, compared to 13% in the control group (odds ratio 1.6).

The common shortcoming of the above three studies is they only focus on one state. The results therefore may not be generalized to the whole population. Using Pregnancy Risk Assessment Monitoring System data from 15 states, Peterson *et al*. [18] studied how Medicaid coverage of TDT would affect smoking for pregnant women. They find women in states with extensive coverage were 60% more likely to quit smoking than those in states with no coverage. The evidence may be biased because there may have been other state level factors, such as cigarette taxes, anti-smoking sentiment, that are correlated with the level of Medicaid coverage, and also affect smokers’ decisions to quit or not. For example, Medicaid in Utah covers all five forms of medication treatments, but people in Utah also hold strong opinions against smoking. Medicaid in Kentucky does not cover any medication treatment, but Kentucky also has one of the lowest cigarette taxes in the States. Over 70 years, the cigarette tax in Kentucky has only risen by a penny in nominal terms, from $0.02 in 1936 to $0.03 in 2005.

This study includes both state and year fixed effects in the models. The state fixed effects control for constant differences across states that are time-invariant, like anti-smoking sentiment. The year fixed effects account for factors that vary uniformly over time across states.

## 4. Data and Model

**Figure 1.**
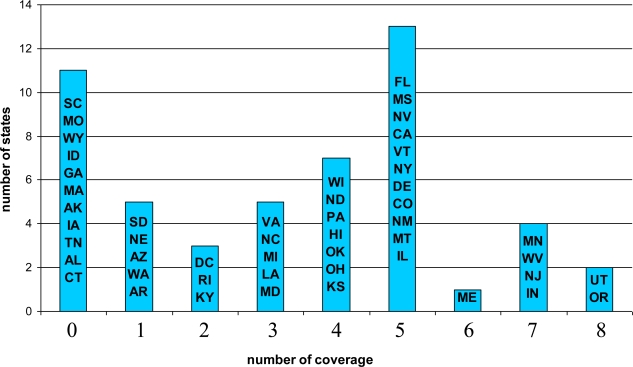
Medicaid Coverage of Tobacco Dependence Treatments in 2002.

The data of TDT coverage by each state’s Medicaid program are from the Centers for Disease Control and Prevention [19]. TDT consists of medication and counseling. Medication includes nicotine nasal spray, nicotine inhaler, nicotine patch, nicotine gum, and Zyban (or generic bupropion). Counseling service includes group, individual, and telephone counseling. In 2005, 42 state Medicaid programs reported offering coverage for at least one form of tobacco-dependence treatment. A TDT coverage index is constructed to measure the extensiveness of coverage. The index is a simple tally of the number of products and programs covered under each state’s Medicaid program. This index varies from 0 to 8. Figure 1 displays the distribution of Medicaid coverage of TDT across states in 2002. Among the 50 states and the District of Columbia in this analysis, 11 states had no coverage of any cessation aids. Tobacco growing states such as Kentucky, Georgia, and Tennessee were less likely to cover medication treatments for Medicaid smokers than other states. Their TDT coverage indices were quite low—0s for Georgia and Tennessee and 2 for Kentucky.

The Current Population Survey—Tobacco Use Supplements (CPS-TUS) is a nationally representative survey and was conducted by the US Census Bureau, sponsored by National Cancer Institute and, since 2001, the Centers for Disease Control and Prevention. The surveys were conducted in September of 1992, 1995, 1998, January and May of 1993, 1996, 1999, June and November of 2001, 2003 and February of 2002, 2003. Each survey provides a sample of over 100,000 individuals aged 15 years and older in a given survey period, with detailed economic and demographic information from respondents. In particular, the data contain information on respondents' cigarette smoking history and current smoking status. CPS March Supplement data provide detailed information on health insurance coverage including Medicaid. In CPS survey, each household is interviewed for four consecutive months, then dropped for eight months, then interviewed again for four more months, then dropped permanently. It is convenient to merge part of Tobacco Usage Supplements with March Supplements using each individual's identification number.

The sample is restricted to those who were Medicaid recipients and were smoking 12 months ago. Those who were not smoking at the survey date are defined as quitting in the past year. After dropping observations with missing values, we have a pooled sample of 5,323 individuals. Respondents of age 15–17 are excluded because NRT is not recommended as a component of pediatric tobacco-use interventions in the 2008 Public Health Service guideline [4]. The definitions and means of all variables are presented in Table 1. The average quitting rate is 9.8%.

The basic econometric model as below:

(1) $$\text{Y}\;=\beta_{0}\;+\;\beta_{1}\text{TDT}\;+\;\beta_{2}\text{Cigarette}\;\text{Tax}\;+\beta_{3}\text{X}\;+\;\beta_{4}\text{T}\;+\;\text{e}$$

Y = 1 if a smoker quits smoking in the past year and Y=0 if she or he continues to smoke. TDT is the tobacco-dependence treatments coverage index that varies across states and years. X is a vector of individual characteristics, including sex, age, age squared, race, household size, family income, education, employment status, marital status and the duration of smoking habits. T includes survey month dummies and year fixed effects.

However, coverage of cessation products is correlated with other state-level characteristics that also affect smoking behavior. For example, Medicaid programs in states with stronger anti-smoking sentiment are likely to cover smoking cessation aids. In order to control for unobserved characteristics across states, state fixed effects (a set of state dummy variables) are included in the model. This method may involve problems of multicollinearity between TDT index and state/year dummies when TDT coverage does not change much in a given state over time. Serious collinearity can lead to very high standard errors of estimated coefficients. The coefficients could have the wrong signs or implausible magnitudes or change dramatically with small changes in data [20]. The Variance Inflation Factor (VIF) is an indicator to detect multicollinearity of an independent variable with other explanatory variables. VIF equals 1/(1-R^2^), where R^2^ is the R-squared for the regression of that independent variable on all the other independent variables. There is no formal result that proves when a VIF is too big, but as a common rule of thumb, a value of VIF above 10 suggests that multicollinearity will present problems [21]. In our sample, a regression of TDT coverage on state dummies and year dummies for years yields an R^2^ of 0.92. Thus, the VIF for TDT coverage is 12.5, which suggests multicollinearity is potentially a serious problem. In addition, the inclusion of state fixed effects accounts for any state-specific factor that is constant. Since anti-smoking sentiment may change over time, the use of state fixed effects may not completely account for unobserved determinants of smoking. As a remedy, a state anti-smoking sentiment index [22,23] is used to replace the state fixed effects in an alternative model.

The key independent variable is TDT. The coefficient on this term will tell us for those covered by Medicaid, how the treatment coverage would affect smoking cessation. The sample is divided into four groups based on age and gender: women aged 18–44; women aged 45 or older; men aged 18–44; men aged 45 or older. Separate regressions are run for each group. Previous studies have found that young adult smokers were most likely to attempt to quit [24] and females were more likely to use smoking cessation aids in a quit attempt [25]. Therefore, it is reasonable to think that TDT coverage might work differently for different groups of people by age and gender.

## 5. Results and Discussion

### 5.1. Descriptive Results

CPS-TUS 2003 data provide information about methods used to try to quit smoking. Table 2 reports the usage of TDT in the past year among Medicaid recipients. Column 1 reports, among those who used TDT, how many of them lived in a state covering TDT. Column 2 and 3 report how many Medicaid smokers used TDT in the past year, only Column 2 relating to states covering TDT, while Column 3 relating to states not covering TDT. Medicaid recipients who used medication to quit smoking were more likely to live in a state where Medicaid program covered that medication. On average, among Medicaid recipients who used medication as cessation aids, more than two-thirds lived in states where the medication was covered by Medicaid. Such a pattern was not seen among counseling users who sought cessation aids—only 18.2% users lived in states where such counseling was covered by Medicaid. One possible reason is that the counseling questions asked in CPS survey may not match to the services covered by Medicaid. Column 2 and 3 suggest that smokers living in a state covering medication are more likely to use it, with 19.3% having used this aid, while only 13.6% Medicaid smokers living in states not covering TDT used the aid. This result is consistent with previous study [26].

### 5.2. Multivariate Regression Results

Tables 3 reports the probit model results for different groups from three specifications, namely the baseline specification (Baseline), the state fixed effects specification (State FE) which includes all the independent variables from Baseline plus state dummies, and the anti-smoking sentiment specification (Sentiment) which includes all the independent variables from Baseline plus the anti-smoking sentiment index. If individual disturbances are correlated within a state, the usual estimates of standard errors will be biased downward. Therefore, robust standard errors adjusted for clustering at the state level are used to address this problem. Only estimates of TDT are presented in Table 3, as TDT is the main concern of this study, but models actually control for all the variables listed in Table 1. The full results are available upon request.

TDT is statistically significant for women of age 18–44 across all three specifications. The results suggest that among female Medicaid smokers aged 18–44, those having higher level of coverage of cessation products were more likely to quit smoking. The marginal effect of Sentiment specification suggests that, if a state Medicaid program covers one more form of TDT, the smoking cessation probability would increase by 0.7 percentage points, or a 7% increase in the baseline cessation rate. However, TDT is not statistically significant for any other group.

The effects of anti-smoking sentiment are as expected. The positive coefficient indicates smokers are more likely to quit in states with high anti-smoking sentiment than in states with low anti-smoking sentiment.

### 5.3. Robustness Check

One explanation of why the effect of TDT coverage on cessation is only significant for women of 18–44 years of age is that many women in this age group are enrolled in Medicaid program because of pregnancy. In ten states in 2000, TDT coverage by Medicaid was for pregnant smokers only [27]. They quit smoking under the concerns of adverse health effect on their babies and risk of spontaneous abortion [28–30]. A report by National Center for Chronic Disease Prevention and Health Promotion [31] said “Compared with women who do not smoke, women who smoke prior to pregnancy are about twice as likely to experience a delay in conception and have approximately 30% higher odds of being infertile; women who smoke during pregnancy are about twice as likely to experience premature rupture of membranes, placental abruption, and placenta previa during pregnancy.” Pregnant smokers are likely to be advised by health professionals to quit as early as possible [32].

CPS-TUS data do not directly provide information on pregnancy. However, if a woman had a child under age 1 in March 2004, then she must be pregnant in March 2003. As discussed before, part of CPS survey respondents in March 2003 can be linked to March 2004. Therefore, we may control for whether a woman was pregnant or not for a subset of the sample. The regression results are reported in Table 4. The estimated coefficient on TDT coverage is still positive and statistically significant at 10% level. As the subgroup sample size is only one forth of the original one, the standard errors are greater in this model. Being pregnant increases the probability of quitting smoking among women aged 18–44 by 13 percentage points.

### 5.4. Effect of TDT Coverage on Initiation

One might think that TDT coverage might make cessation relatively easy and thus induce non-smokers to try smoking. Therefore, smoking initiation might increase because of this moral hazard. This paper also examines the possible effect by estimating smoking initiation models, which are similar to cessation models hereinbefore and use the same data, only the dependent variable Y to be 1 if a respondent initiated smoking in the past 12 months. Table 5 presents the results. TDT coverage is negative and statistically significant for women aged 18–44 in all three specifications. So contrary to what one might think, TDT coverage reduces the possibility of smoking initiation for non-smokers especially for women in the age of 18–44. Probably this is because when health professionals and social workers inform the Medicaid recipients about the coverage of cessation aids, they would also talk about the adverse health consequences of smoking. Therefore, not only more smokers quit but also fewer non-smokers start smoking. TDT coverage is not statistically significant for other three groups.

## 6. Conclusions and Discussions

This study finds evidence that state Medicaid coverage of TDT has a positive impact on smoking cessation for women aged 18–44. For one more form of coverage, cessation rate will increase by 7%. Even when pregnancy is controlled for in the model, the effect still exists. The results are consistent with a recent study by Peterson *et al*. [18], who find women in states with extensive coverage of cessation aids had 18% higher probability of quitting compared to women in states with no coverage. There is no evidence that such effect exists for men or older women.

There are several reasons to explain why TDT coverage may not lead to significant smoking cessation for men and older women. First, smokers interested in quitting may be not aware of that they can obtain financial assistance for tobacco-dependence treatments from Medicaid, as several studies find that a majority of Medicaid smokers are unaware of the program benefit providing coverage for TDT [15,33,34]. Second, most quit attempts are made without pharmaceutical cessation aids. CPS-TUS 2003 suggests only 20% smokers ever used NRT in a quit attempt in the past year. Third, many smokers do not use medications because of safety concerns [6]. Finally, Medicaid may limit the number of courses of medication a person can obtain in a given time period, which may deter smokers from making multiple quit attempts [16].

The central implication of this study is that, simply changing the insurance coverage alone is not sufficient to substantially reduce smoking among Medicaid recipients. More work is needed to improve successful quitting among Medicaid smokers besides providing coverage of pharmaceutical therapies and counseling. Medicaid social workers should make efforts to inform the beneficiaries of the smoking-cessation treatment coverage. Medicaid programs in each state might consider providing more educational materials to help smokers understand the risks and benefits of NRT products, to improve smokers’ knowledge about the safety and efficiency of these medications.

## Figures and Tables

**Table 1.** Definitions and means of all variables.

**Variable**	**Whole sample**	**Women Age 18–44**	**Women Age 45+**	**Men Age 18–44**	**Men Age 45+**
Quit	0.098	0.102	0.101	0.087	0.098
TDT coverage (Tobacco Dependence Treatment)	1.916 (2.38)	1.712 (2.31)	2.155 (2.46)	1.852 (2.34)	2.250 (2.47)
Age	41.464 (15.45)	30.913 (7.14)	58.364 (9.92)	32.560 (7.66)	57.644 (9.86)
Family income (in 1,000 dollars)	13.894 (13.63)	13.660 (13.02)	12.486 (12.92)	15.784 (15.05)	14.601 (14.53)
Household size	2.960 (1.74)	3.564 (1.59)	2.035 (1.46)	3.290 (1.83)	2.139 (1.52)
Number of years smoked	24.648 (14.99)	14.806 (7.39)	39.284 (10.94)	16.771 (8.19)	41.229 (10.89)
Married	0.272	0.221	0.198	0.403	0.378
Employed	0.275	0.349	0.128	0.372	0.160
Female	0.674	1	1	0	0
Race					
• White (omitted)	0.708	0.699	0.718	0.734	0.710
• Black	0.169	0.186	0.178	0.114	0.148
• Hispanic	0.063	0.056	0.056	0.074	0.078
• Others	0.060	0.059	0.048	0.078	0.064
Education					
• Less than high school (omitted)	0.398	0.346	0.461	0.390	0.458
• High school	0.370	0.411	0.316	0.383	0.319
• Some college	0.197	0.225	0.171	0.190	0.167
• College +	0.035	0.018	0.052	0.037	0.056
Year					
• 1993 (omitted)	0.203	0.238	0.162	0.207	0.152
• 1996	0.215	0.224	0.210	0.230	0.182
• 1999	0.174	0.162	0.190	0.167	0.196
• 2001	0.035	0.031	0.031	0.034	0.048
• 2002	0.164	0.151	0.171	0.152	0.208
• 2003	0.209	0.194	0.236	0.210	0.214
Number of observations	5323	2450	1139	862	872

Notes: Standard deviations of continuous variables are in parentheses.

**Table 2.** Usage of TDT in the past year among Medicaid recipients, CPS-TUS 2003.

**TDT usage**	**1**	**2**	**3**	N *
**Medication**				
- Nicotine gum	60.9%	8.2%	7.3%	69
- Nicotine patch	68.0%	19.3%	13.6%	150
- Nicotine nasal spray	87.5%	1.4%	1.3%	8
- Nicotine inhaler	60.0%	3.0%	2.6%	25
- Zyban, Buproprion, Wellbutrin	84.1%	9.0%	4.7%	69
Total	69.8%			321
**Counselling**				
- Telephone	5.9%	1.0%	2.0%	17
- Group	11.8%	1.2%	2.1%	17
- Individual	33.3%	2.6%	2.3%	21
Total	18.2%			55

Notes: N is the number of observations.

**Table 3.** Probit model of smoking cessation.

	**Baseline**	**State FE**	**Sentiment**	**Baseline**	**State FE**	**Sentiment**
Female	Age 18–44			Age 45+		
TDT coverage	0.009*** (0.004)	0.007* (0.004)	0.007** (0.003)	0.003 (0.005)	0.007 (0.006)	0.003 (0.005)
Anti-Smoking sentiment			0.126*** (0.045)			0.015 (0.064)
N	2,450			1,139		
Male	Age 18–44			Age 45+		
TDT coverage	0.003 (0.005)	−0.005 (0.010)	0.001 (0.005)	−0.002 (0.006)	−0.011 (0.010)	−0.002 (0.006)
Anti-Smoking sentiment			0.067* (0.038)			−0.008 (0.058)
N	862			872		

Notes: The table lists marginal effects, with standard errors in parentheses. Statistical significance (based on a two-tailed test) is indicated with asterisks:

*** P < 0.01,

** P < 0.05,

* P < 0.1. N is the number of observations.

**Table 4.** Probit model of smoking cessation, age 18–44 women, controlling for pregnancy.

	**Baseline**	**State FE**	**Sentiment**
TDT coverage	0.015** (0.008)	0.008 (0.013)	0.011* (0.006)
Pregnant	0.129*** (0.051)	0.139** (0.070)	0.129*** (0.051)
Anti-Smoking sentiment			0.180** (0.079)
N		694	

Notes: The table lists marginal effects, with standard errors in parentheses. Statistical significance (based on a two-tailed test) is indicated with asterisks:

*** P < 0.01,

** P < 0.05,

* P < 0.1. N is the number of observations.

**Table 5.** Probit model of smoking initiation.

	**Baseline**	**State FE**	**Sentiment**	**Baseline**	**State FE**	**Sentiment**
Female	Age 18–44			Age 45+		
TDT coverage	−0.004** (0.002)	−0.005** (0.002)	−0.003** (0.001)	0.0002 (0.001)	0.001 (0.001)	0.0002 (0.001)
Anti-Smoking sentiment			−0.018 (0.017)			−0.014 (0.010)
N	5,469			5,250		
Male	Age 18–44			Age 45+		
TDT coverage	−0.001 (0.002)	0.001 (0.004)	−0.001 (0.002)	0.0006 (0.001)	−0.0006 (0.003)	0.0005 (0.001)
Anti-Smoking sentiment			−0.013 (0.019)			0.008 (0.011)
N	1,658			2,209		

Notes: The table lists marginal effects, with standard errors in parentheses. Statistical significance (based on a two-tailed test) is indicated with asterisks:

*** P < 0.01,

** P < 0.05,

* P < 0.1. N is the number of observations.
